# Transformation of Compatible Mating-Type Genes in Monokaryons Triggers Fruiting Body Development by Activating Mating Pathways in *Pleurotus eryngii*

**DOI:** 10.1128/spectrum.05272-22

**Published:** 2023-03-14

**Authors:** Junjun Shang, Sijia Xu, Lihua Tang, Ruiheng Yang, Ming Gong, Yan Li, Ying Wang, Gen Zou, Jianing Wan, Dapeng Bao

**Affiliations:** a Shanghai Key Laboratory of Agricultural Genetics and Breeding, Institute of Edible Fungi, Shanghai Academy of Agricultural Sciences, Shanghai, China; b College of Food Science, Shanghai Ocean University, Shanghai, China; Agroscope

**Keywords:** fruiting body development, *Pleurotus eryngii*, monokaryons, mating-type genes, RNA sequencing

## Abstract

Fruiting body formation is the most important developmental event in the edible mushroom life cycle; however, the genetic regulation of this process is not well understood. *Pleurotus eryngii* is a widely cultivated mushroom with high economic value. The mating of two monokaryons carrying compatible A and B mating-type genes is required for the development of fruiting bodies in *P. eryngii*. In this study, we showed that the monokaryons of *P. eryngii* transformed with compatible homeodomain (A mating type) and pheromone (B mating type) genes can complete fruiting body development but cannot form basidiospores. Transcriptional analyses revealed that expression of endogenous homeodomain and pheromone receptor genes and mating signaling pathways were activated by transferred homeodomain and pheromone genes in the transformants. Our findings provide a novel model for studying fruiting body development, which may accelerate the genetic breeding of edible mushrooms in the future.

**IMPORTANCE** Fruiting bodies of edible mushrooms have high nutritional value. However, the fruiting body development of mushrooms is not well understood, and thus, many wild edible mushrooms of economic importance cannot be cultivated artificially. Moreover, variety among cultivatable mushrooms has improved marginally. Under natural conditions, fruiting body development can be initiated only in a dikaryon, the sexual mycelium obtained from mating two compatible monokaryons. The present work showed induction of fruiting body development in *Pleurotus eryngii* monokaryons by genetic manipulation. Gene expression analyses revealed key genes and signaling pathways involved in the fruiting body development of *P. eryngii*.

## OBSERVATION

The cultivation of edible mushrooms is a sustainable industry that provides food with high nutritional value while reducing traditional agricultural waste (1). Pleurotus eryngii, commonly known as the king oyster mushroom, is one of the most popular edible mushrooms, owing to its large size, meaty texture, good flavor, and long shelf life (2). It is commercially cultivated worldwide, especially in China, where its production is rapidly growing (3, 4). The genetic manipulation of traditional crops, such as corn and rice, has greatly improved their yield and quality. Although mushrooms are new and high-value crops, the genetic traits of most cultivated varieties are not very different from those of their wild relatives and have great potential for improvement (5). The study of fruiting body development can help us understand the genetic mechanisms that control the high productivity and quality of mushrooms.

*P. eryngii* is a tetrapolar basidiomycete with fruiting bodies that produce basidiospores, which germinate to form monokaryotic mycelia. Two compatible monokaryotic mycelia fuse to form a dikaryotic mycelium. Dikaryotic mycelia can be differentiated by the presence of clamp connections, which are hook-like structures present on hyphal cells, and by their potential for fruiting body development (6). Studies on the model basidiomycete Coprinopsis cinerea have shown that the compatibility of monokaryotic mycelia is controlled by two distinct mating-type loci, *matA* and *matB*, located on different chromosomes. Genes on the *matA* locus encode two types of homeodomain transcription factors (*HD* genes), which control the pairing of the two parental nuclei and induce clamp cell formation in the dikaryon. Meanwhile, genes on the *matB* locus encode lipopeptide pheromones and pheromone receptors (*PR* genes), which facilitate septum dissolution and nuclear migration. Products encoded by different *matA* or *matB* alleles on compatible monokaryotic mycelia interact to initiate sexual development (7).

The dikaryon is the persistent vegetative phase of most basidiomycete fungi and contains two unfused nuclei in one cell that can be segregated into two monokaryons by using a protoplast isolation method (8). We previously obtained the monokaryon strains 181 and 183 from the commercially cultivated *P. eryngii* dikaryon strain Xinghan. Sequencing and annotation of the genome of strain 183 contributed to the understanding of the genetic structure of the mating-type loci in *P. eryngii* (9). Using the polyethylene glycol-mediated transformation method (10), we then introduced a DNA fragment containing compatible homeodomain and pheromone genes from monokaryotic strain 181 into the genome of the compatible monokaryotic strain 183, to obtain monokaryotic transformants with clamp connections (11). As the presence of a clamp connection is a typical phenotype of dikaryon, we speculate that the transformants can potentially develop fruiting bodies, as previously reported for monokaryotic mating-type transformants in *C. cinerea* (12, 13).

**FIG 1:**
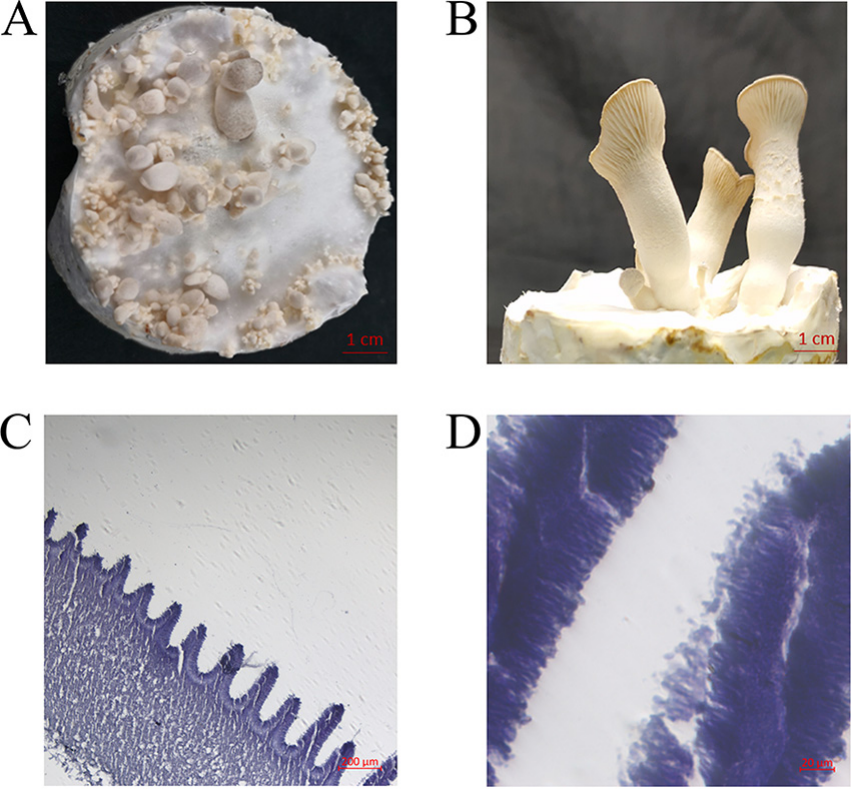
Fruiting bodies developed in the monokaryotic transformant T9 with compatible mating-type genes. (A) The primordia of the transformant T9. (B) The fruiting bodies of the transformant T9. (C and D) Sectioning and microscopic observation of the gills revealed a lack of spore formation in the fruiting bodies of the transformant T9.

To test this speculation, nine different monokaryotic transformants with clamp connections were fruited in sawdust medium under ideal environmental conditions (6). The parental dikaryon strain Xinghan and monokaryon strain 183 were used as positive- and negative-control strains, respectively. The Xinghan strain developed full fruiting bodies and dispersed spores in 60 days after inoculation, whereas strain 183 did not develop any fruiting bodies. All nine transformants developed primordia (the primordia of transformant T9 are shown in Fig. 1A) and fruiting bodies with caps, stalks, and gills (the fruiting bodies of transformant T9 are shown in Fig. 1B). The fruiting bodies of transformants were relatively small, and their development was delayed by more than 40 days compared to that of the Xinghan strain. Moreover, the fruiting bodies of the transformants did not disperse spores. Sectioning and microscopic observation of the gills revealed a lack of spore formation in the fruiting bodies of the transformants (the micrographs of transformant T9 are shown in Fig. 1C and D). These results indicated that the development of fruiting bodies and spore formation are two separate processes in *P. eryngii*. The transformation of compatible mating-type genes triggered the development of fruiting bodies in monokaryons but did not lead to meiosis or spore formation in *P. eryngii*. Similar observations have been previously published for *C. cinerea* (12, 13). Transformation of *matA* genes alone could, in some instances, induce fruiting; however, the development of basidiospores was blocked, prior to karyogamy in the basidia (12). This changed in a subset of transformants, which in addition received compatible *matB* genes by cotransformation with *matA* genes (13). These data suggested that in *C. cinerea*, fruiting body initiation and induction of karyogamy, meiosis, and basidiospores are controlled by two different regulatory schemes, with the latter triggered by adequate expression of compatible *matB* genes.

To analyze the gene expression patterns of transformants with compatible mating-type genes, we performed RNA sequencing (RNA-seq) and compared the transcriptomes of the wild-type monokaryon strain 183 (sample marked X183), wild-type dikaryon strain Xinghan (sample marked XH), and three independent transformants (samples marked T4, T7, and T9). Mycelia were grown at 25°C in the dark on plates containing YMG medium (1% glucose, 1% malt extract, and 0.4% yeast extract) (10). Total RNA was extracted from the mycelia grown on plates without induction of fruiting of the five strains. Samples were obtained from three biological replicates of each strain. RNA-seq was performed using the Illumina HiSeq platform. Reads were mapped to the reference genome of strain 183 (GenBank accession no. MAZY00000000), and gene expression levels were calculated as fragments per kilobase per million reads. The DESeq R package was used to identify differentially expressed genes (DEGs) across the samples (14). Significant gene expression differences were assessed using a threshold for fold change (|log_2_ fold change| > 1) and false-discovery rate (*P < *0.05).

**FIG 2:**
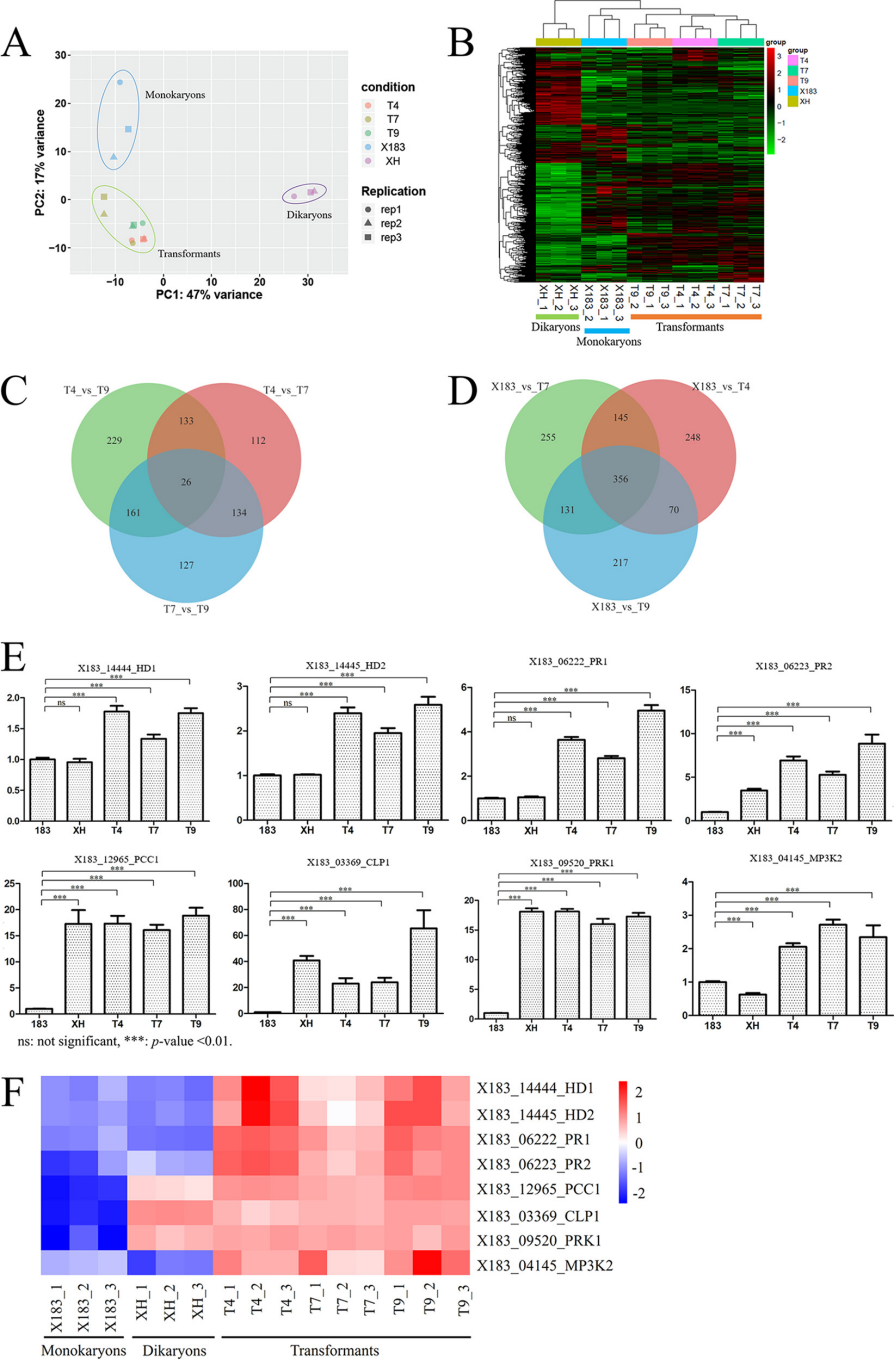
RNA sequencing and qRT-PCR analyses. (A) Principal-component analysis of the transcriptomes. (B) Heatmap clustering of the differentially expressed genes. (C) Venn diagram of the DEGs identified between three independent transformants. (D) Venn diagram of the DEGs identified between the wild-type monokaryotic strain and each of the three transformants. (E and F) qRT-PCR (E) and heatmap (F) analysis results based on the RNA-seq data revealed that the mating-type genes and key genes in the mating-type signaling pathway were induced in the transformants.

The genome-wide gene expression levels of the samples are listed in Table S1 in the supplemental material. Principal-component analysis of the RNA-seq data revealed that the samples from the three transformants (each prepared in triplicate) exhibited similar expression patterns and could be clearly distinguished from the wild-type monokaryon and dikaryon samples (Fig. 2A).

We performed pairwise comparisons of the gene expression levels of all samples. A total of 3,406 DEGs were identified among the 14,445 annotated genes from the reference genome of strain 183 (Table S2). Heatmap clustering of the DEGs showed that the gene expression patterns of the transformant samples T4, T7, and T9 were more similar to those of the monokaryotic sample X183 than to those of the dikaryotic sample XH (Fig. 2B). There were 1,707 DEGs between X183 and the dikaryotic sample XH, whereas there were 821, 888, and 776 DEGs between X183 and the transformants T4, T7, and T9, respectively, which were only approximately half of the number of DEGs between X183 and XH. In pairwise comparisons of the transformant samples, we detected 405 DEGs between samples T4 and T7, 549 DEGs between samples T4 and T9, and 448 DEGs between samples T7 and T9. Only 26 common DEGs were detected among the three pairwise comparisons (Fig. 2C), indicating that the DEGs among transformants were likely to have been caused by random insertions of the foreign DNA fragment. Among the DEGs identified between the wild-type monokaryotic strain and each of the three transformants, 356 genes were common (Fig. 2D). We speculate that these DEGs were affected by the transferred mating-type genes and are important for the development of fruiting bodies in *P. eryngii*.

To detect the biological processes that control fruiting body development in the transformants, gene ontology (GO) (15) and Kyoto Encyclopedia of Genes and Genomes (KEGG) (16) analyses were performed for the DEGs identified between the wild-type monokaryon and transformants. GO analysis revealed that mating-type factors and G-protein-coupled receptors were enriched in the transformants (Fig. S1), and KEGG analysis showed an enrichment of genes involved in mitogen-activated protein kinase (MAPK) signaling in the transformants (Fig. S2). Because the MAPK signaling is crucial for mating in fungi (17, 18), we believe that mating-type pathways were activated in the transformants.

We used quantitative reverse transcription-PCR (qRT-PCR) analysis to verify the expression differences of the mating-type genes and key genes in the mating-type signaling pathway identified using RNA-seq. RNA isolation and qRT-PCR were performed as previously described (19), and relative gene expression levels were calculated using the 2^–ΔΔ^*^CT^* method (20). The primers used for qRT-PCR are listed in Table S3. The qRT-PCR results (Fig. 2E) were highly consistent with the RNA-seq data (Fig. 2F). Both RNA-seq and qRT-PCR analyses showed that two endogenous *HD* genes (*X183_14444* and *X183_14445*) and two endogenous *PR* genes (*X183_06222* and *X183_06222*) were upregulated in the transformants, confirming the successful transformation of the *HD* and *PR* genes in the transformants.

In *Coprinopsis cinereus*, the genes *clampless* (*clp1*) (21) and *pseudoclamp connection formation* (*pcc1*) (22) regulate clamp cell development and are considered key genes in the *matA* signaling pathway, downstream of *HD* genes (23). Our qRT-PCR and RNA-seq analyses revealed that the expression of the *clp1* homolog, *X183_03369*, was dramatically upregulated in the three transformants, compared to the wild-type monokaryotic strain. Similarly, the expression of the *pcc1* homolog, *X183_12965*, was strongly induced compared to the monokaryotic strain.

In Saccharomyces cerevisiae, pheromones bind to their receptors and activate the serine/threonine kinase Ste20p, which triggers activation of the MAP kinase cascade to transduce the pheromone signal (18). Both the RNA-seq and qRT-PCR data showed that the expression of the serine/threonine kinase *X183_09520* and MAP kinase *X183_04145* were upregulated in the transformants compared to the wild-type monokaryon. Because key homologous genes in the *matA* and *matB* signaling pathways of model fungi were induced in the transformants, we conclude that the mating signaling pathway was activated in *P. eryngii* monokaryons transformed with compatible mating-type genes.

### Data availability.

The sequenced raw reads data have been deposited in the Sequence Read Archive database under BioProject accession number PRJNA899321.
