# Supplementary material for: Transformation of Compatible Mating-Type Genes in Monokaryons Triggers Fruiting Body Development by Activating Mating Pathways in Pleurotus eryngii
Source: Microbiol Spectr. 2023 Mar 14;11(2):e05272-22. doi: 10.1128/spectrum.05272-22 (PMC10100773; doi:10.1128/spectrum.05272-22)
Supplement: Supplemental file 4 — Supplemental material. Download spectrum.05272-22-s0004.pdf, PDF file, 0.07 MB [file spectrum.05272-22-s0004.pdf]

Supplemental legends:

Figure S1. Gene ontology (GO) analyses of the DEGs between the monokaryon strain 183 and transformant T7.

Figure S2. Kyoto Encyclopedia of Genes and Genomes (KEGG) analyses of the DEGs between the monokaryon strain 183 and transformant T7.

Table S1. Genome-wide gene expression levels of the samples.

Table S2. Differentially expressed genes (DEGs) among the samples.

Table S3. Primers used for qRT-PCR.

Figure S1

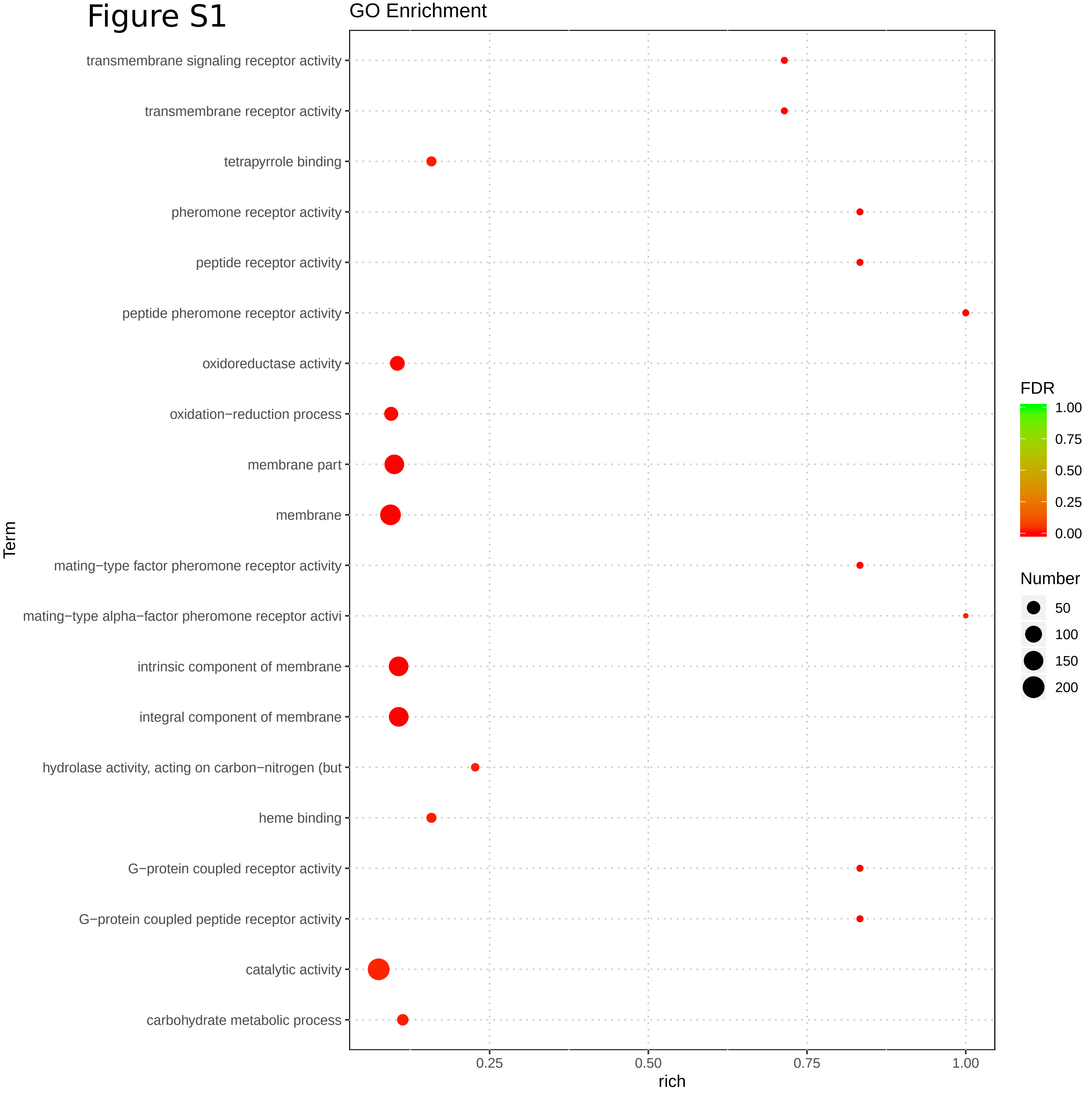
GO Enrichment
Term
transmembrane signaling receptor activity
transmembrane receptor activity
tetrapyrrole binding
pheromone receptor activity
peptide receptor activity
peptide pheromone receptor activity
oxidoreductase activity
oxidation−reduction process
membrane part
membrane
mating−type factor pheromone receptor activity
mating−type alpha−factor pheromone receptor activi
intrinsic component of membrane
integral component of membrane
hydrolase activity, acting on carbon−nitrogen (but
heme binding
G−protein coupled receptor activity
G−protein coupled peptide receptor activity
catalytic activity
carbohydrate metabolic process
0.25
0.50
0.75
1.00
rich
FDR
1.00
0.75
0.50
0.25
0.00
Number
50
100
150
200

Figure S2

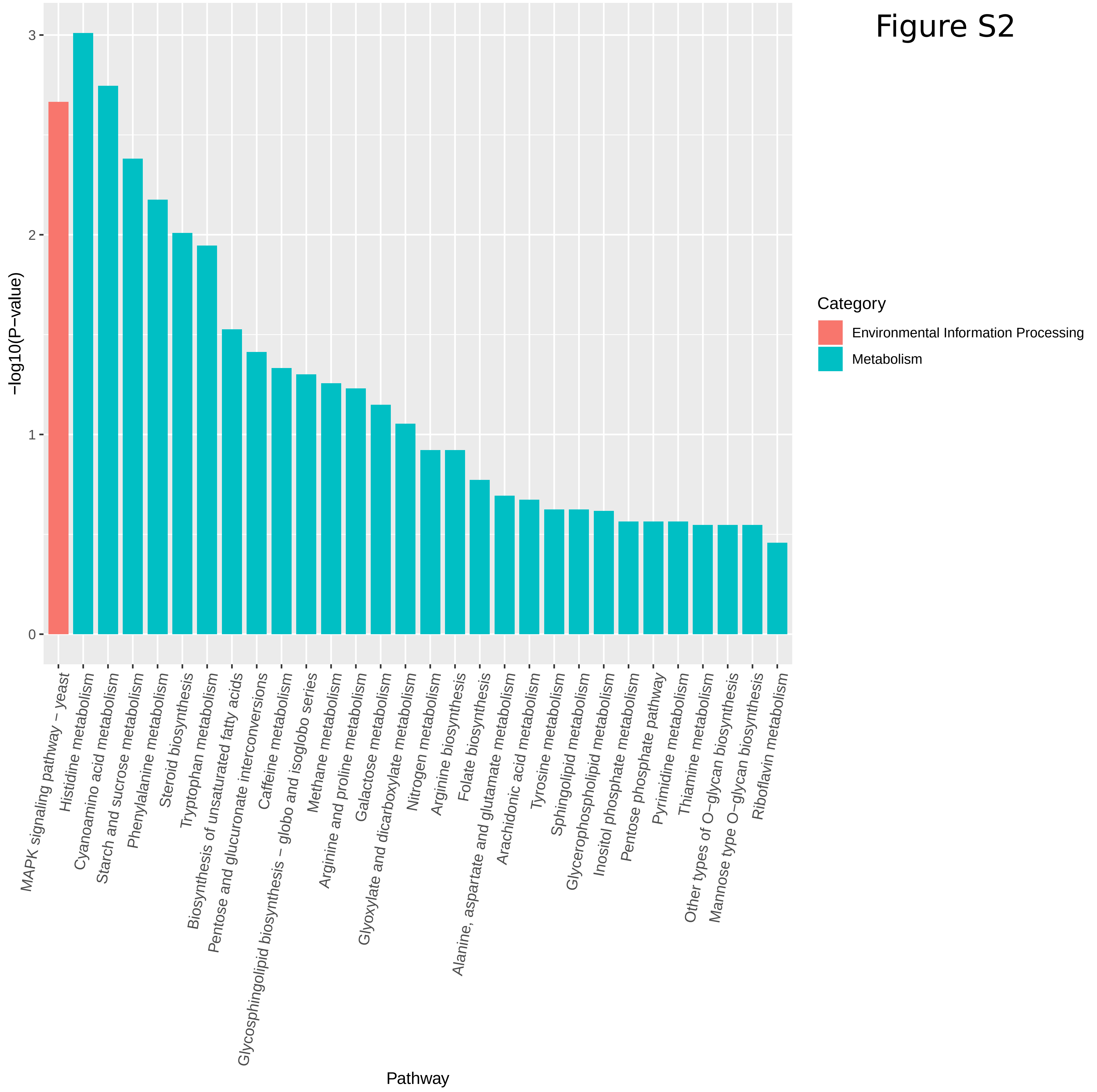

−log10(P−value)
0
1
2
3
Pathway
MAPK signaling pathway − yeast
Histidine metabolism
Cyanoamino acid metabolism
Starch and sucrose metabolism
Phenylalanine metabolism
Steroid biosynthesis
Tryptophan metabolism
Biosynthesis of unsaturated fatty acids
Pentose and glucuronate interconversions
Caffeine metabolism
Glycosphingolipid biosynthesis − globo and isoglobo series
Methane metabolism
Arginine and proline metabolism
Galactose metabolism
Glyoxylate and dicarboxylate metabolism
Nitrogen metabolism
Arginine biosynthesis
Folate biosynthesis
Alanine, aspartate and glutamate metabolism
Arachidonic acid metabolism
Tyrosine metabolism
Sphingolipid metabolism
Glycerophospholipid metabolism
Inositol phosphate metabolism
Pentose phosphate pathway
Pyrimidine metabolism
Thiamine metabolism
Other types of O−glycan biosynthesis
Mannose type O−glycan biosynthesis
Riboflavin metabolism
Category
Environmental Information Processing
Metabolism
